# Development and Reliability and Validity Test to the Parenting Stress Questionnaire for Two-Child Mothers

**DOI:** 10.3389/fpsyg.2022.850479

**Published:** 2022-05-04

**Authors:** Zhinuo Zhang, Yulong Tang, Xiyue Chen, Xinyi Lin, Jiaheng Tao

**Affiliations:** Institute of Applied Psychology, College of Education, Zhejiang University of Technology, Hangzhou, China

**Keywords:** two-child mother, parenting stress, developing questionnaire, China, validity

## Abstract

China is getting old before it gets rich. Among women of childbearing age, there seems to be little interest in having multiple children, and parenting stress may be one of the reasons. There are differences in the parenting stress felt by mothers with one child and those with two, but there is no questionnaire specifically aimed at the parenting stress felt by mothers of multiples in China. The purpose of the present study is to develop and verify a questionnaire specifically aimed at measuring the stress of two-child mothers in the Chinese context. We chose mothers as participants who were younger than 50 years old and their second child were younger than 18 years old as participants. The initial questionnaire was created after analyzing the results of 83 participants’ open questionnaires and 16 participants’ qualitative interviews. Item analysis and exploratory factor analysis were conducted with 279 participants. The final questionnaire was created after conducting reliability and validity tests on the responses of 263 participants to 23 items on the questionnaire covering four factors: characteristics of mother, environmental factor, characteristics of child, and relationship between the two siblings. The results of confirmatory factor analysis indicated that the four-factor model fit well (χ^2^/*df* = 2.00, CFI = 0.91, TLI = 0.90, SRMR = 0.06, RMSEA = 0.06). McDonald’s omega coefficients and split-half reliability coefficients both ranged from 0.50 to 0.95. The questionnaire scores were significantly positively correlated with parental burnout, the regret of having a second child and parenting stress, and were significantly negatively correlated with the intention of having a third child and support for the three-child policy. Overall, the present study confirmed the reliability and validity of the parenting stress questionnaire for two-child mothers, which can be used to measure the parenting stress experienced by mothers of multiples in China.

## Introduction

China is aging (Bai and Lei, 2020). In the face of successive declines in the birth rate, China has repeatedly reformed restrictions on family size (Gietel-Basten et al., 2019). From the one-child policy to the current three-child policy, the continuous loosening of policies has reflected the government’s encouragement of fertility, but these policies have clearly been at odds with the willingness of parents to have more children for a long time (Zeng and Hesketh, 2016; Chen, 2021). As the main child-bearer and caregiver of children (Cheung, 2000), mothers experience the greatest parenting stress, which may be an important factor inhibiting the willingness to have more than one child (Li et al., 2017; Chen et al., 2019; Jiang and Liu, 2020). Due to the long-term implementation of the one-child policy in China, mothers usually lack the experience of raising two children. As what expected, two-child mothers in China experience higher parenting stress than one-child mothers (Hong and Liu, 2021; Qian et al., 2021b). In addition, existing studies have shown that there is a difference between the parenting stress of one-child mothers and two-child mothers (Hong and Liu, 2020), the latter of which is complicated by sibling relationships (Tippett and Wolke, 2015; Chen, 2020). However, the current measurement of parenting stress for two-child mothers uses the measurement for one-child mothers’ parenting stress, which may not fully and accurately measure their stress levels. Therefore, it is necessary to systematically understand the parenting stress of two-child mothers and develop appropriate measurement tools.

Parenting stress usually refers to the feelings experienced by parents when they perceive that the needs related to parenting exceed their personal and social resources available to meet these needs (Cooper et al., 2009). The reason why the parenting stress of two-child mothers needs to be watched closely is that during the transition from a one-child family to a two-child family in China, the role of mothers and family relationships have changed. The initial change in the role may make the mother feel unable to cope or meet the needs of caring for two children, thereby affecting the mother’s own mental health and the child’s physical and mental health (Chen, 2020).

A number of studies have explored parenting stress. Belsky (1984) proposes a process model of parenting stress that includes three domains: personal psychological resources of parents, characteristics of the child, and contextual sources. Abidin (1992) found that the main sources of stress were personality of the parent, characteristics of the child and environmental factors, and he emphasized the influence of parent–child characteristics and interactions on parenting stress. Berry and Jones (1995) stated that parental characteristics were the source of parenting stress, and they measured stress by parents’ emotions and role satisfaction in four dimensions: parental rewards, parental stressors, parental satisfaction, and lack of control. The model of determinants of parenting stress showed that the individual characteristics of children and parents, husband and wife relationships, parent–child relationships, environmental characteristics, and the interaction between these factors all play a role in parenting stress (Crnic and Acevedo, 1995). In addition, Östberg and Hagekull (2000) analyzed the parenting stress of mothers from the perspectives of the mother’s factors and external stressors. The results showed that a number of factors were directly related to increased stress: high workload, low social support, perception of the child as fussy-difficult, negative life events, child caretaking hassles, more children in the family, and older maternal age. Although researchers examining the current structure of parenting stress have not reached a consistent conclusion, they report similarities in the division of dimensions and the generalization of content; that is, there are three main aspects involved in parental stress: characteristics of parents, characteristics of children, and environmental factors. The aspect of the environment is mainly composed of family-level factors and society-level factors. For example, parental psychological characteristics, such as personality characteristics, mental health, relationship difficulties, work-family balance, and other factors will directly or indirectly affect parenting stress (Mulsow et al., 2002; Cain and Combs-Orme, 2005). Child factors such as temperament and behavior will also directly affect the level of parenting stress (Jackson, 2000; McBride et al., 2002). Regarding environmental factors, parents’ financial status, intimacy between parents and coparenting processes are also related to parenting stress (Östberg and Hagekull, 2000; Liu et al., 2020; Qian et al., 2021b).

Although there are currently a variety of measurement tools for parenting stress (i.e., Belsky, 1984; Abidin, 1992; Berry and Jones, 1995), there are still some aspects that need improvement and are mainly reflected in the following two aspects. On the one hand, although the same measurement tools are used to measure the parenting stress of mothers with one or more children in foreign countries where family size is not restricted (Krieg, 2007), Chinese family size has been restricted to one child for a long time. Now that the unique one-child policy has been lifted, the parenting stress of mothers in two-child families should receive attention, and their stress should be further distinguished from that of mothers with one child.

On the other hand, as a new family model, two-child families may face different parenting stress than do one-child families. The family crisis model and the stressful life event model point out that the birth of the second child, as a stressful life event, creates more psychological stress for parents (Stewart, 1990). The law of family interaction proposed by Bossard (1945) assumes that the family relationships in a two-child family are more complex and diverse than those in a one-child family. A mother with two children needs to adapt to two children and establish the same basic but independent parent–child relationship with each child. The mother also needs to handle the sibling relationship correctly and smoothly (Chen, 2020). Therefore, compared to one-child families, two-child families may face greater challenges and stress. It is worth noting that the current research on the parenting stress of the two-child family assumes that the parenting stress in one-child and two-child families is homogeneous in structure. However, the structure of two types of families may be heterogeneous (Hong and Liu, 2020). For example, there are differences in child-raising tasks and parent–child interactions (Chen and Shi, 2017). Empirical studies have found that the sibling structure had a significant impact on maternal parenting stress (Qian et al., 2021a). But there are no items that incorporate the sibling relationship in measures of parenting stress.

Overall, it is necessary to formulate a questionnaire that measures the parenting stress of two-child mothers in China. Therefore, this study developed a questionnaire specifically aimed at measuring the stress of two-child mothers through open questionnaires and qualitative interviews. Then, we confirmed the reliability and validity of the parenting stress questionnaire for two-child mothers.

## Materials and Methods

### Participants

In response to the COVID-19, data were collected from a sample of 542 two-child mothers from an online survey questionnaire network^1^ from 24 provinces including Beijing, Zhejiang, Guangdong in the period of COVID-19 pandemic in China. In order to reduce the uncertainty of pressure variation due to the large age gap in mother and the second child, we limited the ages of mother younger than 50 and the second child younger 18 in the study.

### Procedures

Eighty-three participants completed an online questionnaire that was presented as a “Questionnaire for Women on Second-Child Parenting.” To obtain more sources of stress and limit the scope of the survey to compile the outline of the interview, the questionnaire addressed the participants with a single, open-ended question, “What difficulties have you encountered in the process of raising your second child? Please explain, list them, and be as detailed as possible.”

The participants’ testimonies were taken during unstructured interviews, which were recorded and transcribed for the purpose of analysis. Eight participants were selected for the initial interview. After coding their responses and obtaining the preliminary themes, we continued conducting purposive sampling with an additional eight participants until no new themes emerged. The interviews were conducted by a trained research assistant and lasted approximately 30 min. The outline of initial interview in the Supplementary Material.

Data transcribed from the interview recordings were encoded and analyzed using NVivo 12.0 analysis software. After multiple induction summarizing, 45 free codes, 8 correlation codes and 3 core codes were obtained. The associated coding of the behavior and characteristics of children (frequency, 36) and the relationship between the two siblings (frequency, 31) are summarized as the “child factor”; family internal environment (frequency, 91) and external social environment (frequency, 63) as the “environmental factor”; relationship difficulties (frequency, 37), emotional changes (frequency, 25), physical changes (87 frequency, 87), and parenting constraints (frequency, 58) as the “mother factor.” According to the results and the theoretical model of parenting stress (Belsky, 1984; Abidin, 1992), a structural model of parenting stress was constructed around the three main elements: children, environment, and mother.

Based on the thematic analysis of the qualitative interviews and the measurement tools (Abidin, 1992; Östberg and Hagekull, 2000), 26 influential items were identified of the initial questionnaire. These items included mothers’ psychological characteristics, children’s temperament and behavior, the relationship between two siblings, and environmental factors. Finally, two quality monitoring questions were set up to check whether the participants answered the questionnaire carefully: “I regret having a second child” and “I am very willing to continue to give birth.” Items were rated on 5-point Likert scales: 1 (“completely inconsistent”) to 5 (“completely consistent”), with a higher score representing higher parenting stress. Except for the item “I am very willing to continue to have a third child,” the other items were scored positively. The initial questionnaire was collected in the form of online questionnaire, which was used for item analysis and EFA.

In the end, based on the results of the initial questionnaire, the items of the initial questionnaire were modified and deleted to form a formal questionnaire. In addition to the formal questionnaire, some of the participants also needed to complete the revised Chinese version of the parental stress scale or the revised Chinese version of the parental burnout assessment, which was used for the compatibility validity analysis and criterion correlation validity analysis.

### Measures

A revised Chinese version of the Parental Stress Scale (Berry and Jones, 1995) by Cheung (2000) was administered. The scale involves two dimensions: parenting burden and satisfaction and consists of 17 items. On a five-point scale ranging from “1” (strongly disagree) to “5” (strongly agree), items 11–17 are reverse scored; the higher the total score is, the higher the parenting stress. Cronbach’s alpha coefficient of this scale was 0.85.

A revised Chinese version of the Parental Burnout Assessment (Roskam et al., 2018) by Cheng et al. (2020) was used in this study. The assessment consists of 21 items, which are scored on a seven-point scale, ranging from “1” (never) to “7” (daily). The higher the score is, the higher the level of parental burnout. Cronbach’s alpha coefficient of this scale was 0.97.

The survey also included three questions as criteria: “I regret having a second child,” “I am very willing to have a third child,” and “I strongly support the three-child policy.”

### Data Analysis

Before conducting a factor analysis, some questionnaires were invalidated, and respondents were removed from the sample. Reasons for exclusion included a second child who was older than 18, questions left unanswered, irregular or illogical answers and responses given significantly faster than the average time taken by other respondents to complete the survey.

For the purpose of factor analyses, the sample was split into two subsamples of 279 (subsample 1) and 263 participants (subsample 2) to compute exploratory factor analysis (EFA) and confirmatory factor analysis (CFA), respectively. The 542 participants were randomly assigned to one of the two subsamples.

Subsample 1 (*n* = 279) was used for item analysis and EFA (using principal component analysis and Varimax orthogonal rotation). Before factor analysis, we performed Bartlett’s test of sphericity and the Kaiser–Meyer–Olkin measure of sampling adequacy test (KMO test). The method of principal component analysis and varimax rotation were used. These analyses were based on a comparison between eigenvalues from a factor analysis of the actual data and performed using SPSS 22.0. The criterion of eigenvalues > 1 was employed to select components (Munro, 2005). Items with factor loadings that exceeded 0.50 (Hair et al., 2006) and the cross-loaded on two factors with factor loadings less than 0.4 were included (Ferguson and Cox, 1993).

Subsample 2 (*n* = 263) was used for reliability, construct validity, and criterion correlation validity regarding responses to the three questions about regret about having a second child, willingness to have a third child, and support of the three-child policy. Seventy-seven participants were also used for the compatibility validity analysis of the parenting stress scale, and 85 respondents participated in the criterion correlation validity analysis of the parenting burnout scale.

A CFA was then performed on the second subsample (*n* = 263). The measurement model included four latent variables representing the characteristics of mother, environmental factors, characteristics of the child, and relationship between two siblings. There were 9 items for characteristics of mother, 8 for the environment factors, 4 for characteristics of child, and 2 for the relationship between two siblings. Analyses were conducted using the maximum likelihood estimation with Mplus 8.0. Several goodness-of-fit indexes were used to determine the acceptability of the models. In addition to the chi-square model, which is highly sensitive to sample size and leads to model rejection even when the model misspecification is relatively minor (Hayduk, 1996), the root means square error of approximation (RMSEA), the standardized root mean square residual (SRMR), the comparative fit index (CFI), and the Tucker–Lewis index (TLI) were used (Acock, 2013). For CFI and TLI, values close to 0.90 or greater are acceptable to good. RMSEA and SRMR should preferably be less than or equal to 0.06 (Kelley and Lai, 2011). Reliability was estimated with McDonald’s omega coefficients and split-half reliability coefficients.

## Results

### Study Sample

A summary of the demographic characteristics of the three samples is provided in Table 1. Subsample 1 were aged 23.00–48.33, and their firstborns, ranging from 1.33 to 23.58 years of age, and their younger siblings, ranging in from 0.08 to 17.17 years of age. Subsample 2 were aged 20.50–48.83, and their firstborns, ranging from 1.58 to 23.75 years of age, and their younger siblings, ranging in from 0.08 to 17.17 years of age. All sample were aged 20.50–48.83, and their firstborns, ranging from 1.33 to 23.75 years of age, and their younger siblings, ranging in from 0.08 to 17.17 years of age.

**TABLE 1:** Sample characteristics.

	Subsample 1	Subsample 2	Sample
	Mean (*SD*)	Mean (*SD*)	Mean (*SD*)
Mothers’ age (years)	34.70 (6.26)	34.14 (6.23)	34.43 (6.25)
Firstborns’ age (years)	9.32 (5.69)	9.36 (5.82)	9.34 (5.75)
Younger siblings’ age (years)	4.57 (4.13)	4.44 (4.54)	4.51 (4.33)
Age gap between firstborns and their young sibling (years)	4.76 (2.99)	4.92 (3.14)	4.83 (3.06)
Total	279	263	542

### Items Analysis

According to the total score distribution of subsample 1 (*n* = 279), 27% of the high-end and low-end participants were divided into high and low groups, respectively. The results of the independent sample *t test* showed that there were significant differences in the scores of 26 initial items between the two groups, with *t* values ranging from −5.60 to −15.44 and *p* values less than 0.001. The total correlations of items were between 0.4 and 0.75, and the *p* value was less than 0.001 when *r* was greater than 0.4. Finally, reliability analysis was carried out to investigate Cronbach’s alpha coefficient of the total questionnaire. After deleting any one item, the results showed that the Cronbach’s alpha coefficient of the total questionnaire remained between 0.94 and 0.95. In conclusion, each item had good discrimination. The covariance matrix was in Supplementary Table 1.

### Exploratory Factor Analysis of the 26 Items

The data of subsample 1 (*n* = 279) were used for exploratory factor analysis. The KMO value was 0.941, and the value of the Bartlett test of sphericity was χ^2^ (325) = 4111.97, *p* < 0.001. After multiple exploratory factor analyses, 3 items were deleted, and 23 items were retained, resulting in 4 factors, with a cumulative variance explanation rate of 60.36%. Finally, 23 items were retained, resulting in four factors. According to the concept of questionnaire preparation and the meaning of the items, the four factors were named as: ➀ Characteristics of mother, including 9 items, involving mother’s feelings at her own level of the parenting stress, including physical, emotional, parenting constraints, and relationship management; ➁ Environmental factors, including 8 items, related to the parenting stress felt by mothers in the external society and internal family, including the stress on economy, education and care problems; ➂ Characteristics of child, including 4 items, that was, the parenting stress caused by children’s own personality and behavior; ➃ Relationship between two siblings, including 2 items, mainly referred to the parenting stress of two-child mothers in dealing with the relationship and care between two children. The factor load of each item is between 0.51 and 0.83, and the commonality was between 0.37 and 0.76. The items, loadings and commonality of the four-factor structure of the are presented in Table 2.

**TABLE 2:** Loading parameter estimates in EFA from the four-factor solution and commonality estimates for the 26-item version of the questionnaire in subsample 1 (*n* = 279).

Items	CM	EF	CC	RS	Commonality
7. After raising two children, I hardly have time to do what I like	0.76				0.68
10. Raising two children reduced my contact with friends	0.76				0.64
8. Raising two children will make me feel disconnected from society	0.73				0.64
5. Raising two children completely restricts my development at work	0.67				0.55
4. Raising two children makes me easy to lose my temper	0.67				0.58
2. After giving birth to my second child, I felt that I was not interested in many things and did not have the same enthusiasm as before	0.66				0.53
3. Raising two children makes me often feel inexplicably anxious	0.64				0.58
6. My working hours and the time I take care of my two children often conflict	0.62				0.44
1. After giving birth to my second child, my body often feels tired	0.51				0.37
20. After having a second child, the money I spend on myself has decreased significantly		0.80			0.71
24. I often worry about not being able to create good educational conditions for my two children		0.74			0.66
23. The current fierce competition in education makes me anxious about the future of my two children		0.72			0.62
25. I often feel troubled when I need to tutor children		0.72			0.65
18. The daily expenses of raising two children make me feel very stressed		0.69			0.66
26. I often worry about whether the way of educating children is correct		0.65			0.64
19. The expenditure on education for two children makes me very stressed		0.62			0.54
21. I am troubled by the daily problems (such as eating, picking up, getting sick, etc.) when taking care of the two children		0.61			0.56
15. I feel my child doesn’t listen to me			0.81		0.76
14. I think my child is very naughty			0.76		0.68
17. My kids are easy to get angry because of small things			0.66		0.54
13. Maintaining good communication with two children is a problem for me			0.63		0.65
11. I am very concerned about whether the relationship between the two children is harmonious				0.83	0.75
12. I often worry about how to balance the care and care of the two children				0.76	0.76
22. No one can share the pressure of taking care of my children well	0.52	0.41			
Eigenvalue	5.71	5.03	3.17	1.78	
Variance interpretation rate (%)	21.96	19.35	12.19	6.86	

*CM, Characteristics of Mother; EF, Environmental Factor; CC, Characteristics of Child; RS, Relationship between Two Siblings.*

### Confirmatory Factor Analysis

The skewnesses and kurtoses reflect that the data do not falsify the parametric assumptions (Table 3). Hence, CFA could be adopted, i.e., the skewnesses of all the items fall between −2 and 2 while the kurtoses of all the items fall between −7 and 7 (George and Mallery, 2010).

**TABLE 3:** The means, *SD*s, skewnesses, and kurtoses of the items in subsample 2 (*n* = 263).

Item	Means	*SD*	Skewness	Kurtosis
CM 1	4.00	0.86	−0.93	1.03
CM 2	3.65	1.11	−0.60	−0.30
CM 3	3.76	1.07	−0.72	−0.07
CM 4	3.73	1.06	−0.53	−0.43
CM 5	3.61	1.09	−0.42	−0.49
CM 6	3.70	1.05	−0.60	−0.20
CM 7	3.73	1.08	−0.61	−0.37
CM 8	3.48	1.19	−0.29	−0.89
CM 9	3.52	1.12	−0.46	−0.56
RS 1	4.19	0.88	−1.05	0.82
RS 2	3.84	1.02	−0.67	−0.17
CC 1	3.43	1.11	−0.26	−0.68
CC 2	3.22	1.10	−0.02	−0.62
CC 3	2.98	1.17	0.10	−0.75
CC 4	3.10	1.23	−0.14	−1.02
EF 1	3.72	1.08	−0.62	−0.22
EF 2	3.95	1.03	−0.77	−0.12
EF 3	3.97	0.99	−0.87	0.32
EF 4	3.84	1.04	−0.84	0.28
EF 5	3.98	1.02	−1.00	0.73
EF 6	3.97	1.03	−0.99	0.64
EF 7	3.56	1.06	−0.37	−0.41
EF 8	3.98	0.92	−0.90	0.94

*CM, Characteristics of Mother; EF, Environmental Factor; CC, Characteristics of Child; RS, Relationship between Two Siblings.*

Four alternative factor models of subsample 2 (*n* = 263) were tested using CFA. In all of the measurement models estimated, error covariances were fixed to zero, and factors were allowed to correlate. One factor model (M1) was proposed as the null hypothesis, which postulated a single factor on which all the items load. Model 2 (M2) postulated a two-factor structure with correlated factors, similar to the factor solution found in previous studies (Williford et al., 2007). Three factor model (M3) proposed a three-factor structure based on hypothesis at the beginning (Supplementary Figure 1). Table 4 displays the fit indices of the competing models, as well as the model comparisons. Four factor model (M4) provided a better fit to the data, according to the Chi-squared difference, compared with M1 (Δχ2 = 299.24, Δ*df* = 6, *p* < 0.001), M2 (Δχ2 = 164.75, Δ*df* = 5, *p* < 0.001), and M3 (Δχ2 = 66.91, Δ*df* = 3, *p* < 0.001). In addition, in terms of fit indices and parsimony, M4 showed the best fit of CFI, TLI, SRMR, and RMSEA (see Table 4). The standardized solution of the four-factor model showed that all items had a high loading on their factor of characteristics of mother, environmental characteristics of child and relationship between two siblings. All factor loadings were between 0.41 and 0.85.

**TABLE 4:** Fit indices for the estimated models (*n* = 263).

	χ 2	*df*	CFI	TLI	SRMR	RMSEA
M1	747.81	230	0.79	0.50	0.08	0.09
M2	613.32	229	0.84	0.83	0.08	0.08
M3	515.48	227	0.88	0.87	0.08	0.07
M4	448.57	224	0.91	0.90	0.06	0.06

*CFI, comparative fit index; TLI, Tucker–Lewis index; SRMR, the standardized root mean square residual; RMSEA, root mean square error of approximation. Levels ≥0.90 for CFI and TLI, and ≤0.06 for RMSEA and SRMR indicate that the models fit the data well.*

### Reliability Analysis

The data of subsample 2 (*n* = 263) were used for reliability test, including internal consistency reliability (McDonald’s omega, ω) and split half reliability (Spearman-Brown correction). The results showed in Table 5. Except that the reliability index of relationship between two siblings was slightly lower, the reliability index of other factor and total score are more than 0.80, which met the requirements of psychometrics.

**TABLE 5:** Reliability coefficient of parenting stress of two-child mothers (*n* = 263).

	CM	EF	CC	RS	Total
ω	0.90	0.87	0.83	0.56	0.95
Split half reliability	0.93	0.86	0.84	0.50	0.94

*CM, Characteristics of Mother; EF, Environmental Factor; CC, Characteristics of Child; RS, Relationship between Two Siblings.*

### Compatibility Validity

Pearson correlation analysis was conducted to examine the relationships among PSS, the total scores and each factor of questionnaire of two-child mothers and (see Table 6). The results showed that there was a significant positive correlation between PSS and the total score and factors of parenting stress of two-child mothers (except relationship between two siblings), and the correlation coefficient was between 0.32 and 0.91.

**TABLE 6:** Correlation analysis between PSS and total scores and factors of parenting stress of two-child mothers (*n* = 77).

	CM	EF	CC	RS	Total score	PSS
CM	—					
EF	0.83***	—				
CC	0.63***	0.56***	—			
RS	0.58***	0.67***	0.32**	—		
Total score	0.94***	0.94***	0.75***	0.68***	—	
PSS	0.63***	0.53***	0.71***	0.12	0.65***	—
*M*	3.82	3.84	3.31	4.00	3.75	2.89
*SD*	0.74	0.79	0.93	0.84	0.68	0.59

*CM, Characteristics of Mother; EF, Environmental Factor; CC, Characteristics of Child; RS, Relationship between Two Siblings; PSS, Parental Stress Scale.*

***p < 0.001, **p < 0.01.

### Criterion Correlation Validity

The correlation analysis between PBA and the total score and each factor of parenting stress of two-child mothers was shown in Table 7, and the correlation analysis with other efficacy items was shown in Table 8. The results showed that the parenting stress of two-second mothers was significantly positively correlated with parenting burnout and the regret of having a second child. The parenting stress of two-child mothers was significantly negatively correlated with the willingness of having a third child and the support for the three-child policy.

**TABLE 7:** Correlation analysis between PBA and total score and each factor of parenting stress of two-child mothers (*n* = 85).

	CM	EF	CC	RS	Total score	PBA
CM	—					
EF	0.70***	—				
CC	0.56***	0.47***	—			
RS	0.41***	0.56***	0.36**	—		
Total score	0.91***	0.87***	0.74***	0.59***	—	
PBA	0.62***	0.46***	0.66***	0.20^†^	0.65***	—
*M*	3.71	3.85	3.22	3.98	3.70	3.81
*SD*	0.76	0.69	0.99	0.75	0.65	1.37

*CM, Characteristics of Mother; EF, Environmental Factor; CC, Characteristics of Child; RS, Relationship between Two Siblings; PBA, Parental Burnout Assessment.*

***p < 0.001, **p < 0.01, ^†^p < 0.1.

**TABLE 8:** Correlation analysis of the total score of and various factors of two-child mothers with the regret of having a second child, the willingness to have a third child and the support for three-child policy (*n* = 263).

	CM	EF	CC	RS	Total score	RH	WT	ST
CM	—							
EF	0.70***	—						
CC	0.55***	0.48***	—					
RS	0.45***	0.58***	0.26**	—				
Total score	0.91***	0.89***	0.70***	0.61***	—			
RH	0.46***	0.38***	0.51***	0.17**	0.50***	—		
WT	−0.20**	−0.19**	0.02	−0.15*	−0.18**	0.04	—	
ST	−0.19**	−0.12^†^	−0.04	−0.02	−0.14*	−0.12^†^	0.48***	—
*M*	3.70	3.80	3.34	3.91	3.69	2.61	2.22	3.00
*SD*	0.74	0.75	0.88	0.84	0.65	1.24	1.28	1.27

*CM, Characteristics of Mother; EF, Environmental Factor; CC, Characteristics of Child; RS, Relationship between Two Siblings; RH, regret of having a second child; WT, willingness to have a third child; ST, support for three-child policy.*

***p < 0.001, **p < 0.01, ^†^p < 0.1.

## Discussion

This study collected and analyzed the parenting stress of two-child mothers through open questionnaires, which included five problem areas: education, care, physical, economic, and relationship. According to the results of qualitative interviews, we identified three dimensions: mother, child, and environmental. On this basis and referring to previous parenting stress questionnaires, 26 items were included on the initial parenting stress questionnaire. Reliability analysis results showed that McDonald’s omega coefficient for the total questionnaire was 0.95, and the split-half reliability coefficient was 0.94. Except for the relationship between two siblings, McDonald’s omega coefficients and split-half reliability coefficients of all other factors were greater than 0.8.

In exploratory factor analysis, item 16 with factor loadings less than 0.5 was deleted and items 9 and 22 with two factor loadings greater than 0.4 were deleted. This left 23 items based on 4 factors: characteristics of mother, environmental factor, characteristics of child, and relationship between two siblings. On the whole, the three factors of mother, environment and children are consistent with the structure of previous parenting stress theory. The empirical test resulted in the differentiation of child factors into two factors, characteristics of child and relationship between two siblings, showed that the relationship between two siblings was an independent variable that affected the parenting stress of two-child mothers. The 4-factor model obtained by exploratory factor analysis had a clear structure, and the factor load of each item was above 0.50, which could explain 60.36% of the overall variance. The results of confirmatory factor analysis showed that the data of each indicator met the basic requirements of psychometrics, which verified the stability of the questionnaire structure.

In the investigation of compatibility validity, the results of correlation analysis found that, in addition to the relationship between two siblings, the total scores of parenting stress and the scores of various other factors were significantly positively correlated with the PSS score. This may be because the content of PSS does not contain a factor for the relationship between two children, so the impact of this relationship could not be measured.

The correlation validity test was carried out with PBA and the item “regret having a second child” as the criterion. Findings indicated that the total score of parental stress and the scores of various dimensions of two-child mothers were significantly positively correlated with the selected criterion. Selecting the items—willingness to have a third child and support for the three-child policy—as the criteria, the total score of parental stress for two-child mothers was significantly negatively correlated with the selected criterion. The above results indicated that the newly compiled questionnaire for two-child mothers had better correlation validity and could be used as a tool for predicting parenting burnout, the regret of birthing a second child, and willingness to give birth to a third child.

It should be noted that the questionnaire developed in this research has limitations. The questionnaire is mainly aimed at measuring the parenting stress the mothers who have two children, the ages of mothers and their second-child may be an important potential factor affecting the parenting stress of mothers. Therefore, we limit the ages of mothers (less than 50 years old) and second-child (less than 18 years old). However, it is undeniable that the age gap in mothers and their first and second children in the current study is still existence. This may cause the stressors of a mother with an old firstborn are different from those with a small firstborn. And the epidemic may also be a contributing factor to mothers’ parenting stress. The parenting stress of two-child mother was measured in the context of COVID-19, which may lead to greater parenting pressure for mothers, such as isolation or economic pressure caused by the epidemic. In addition, there are only two items about the relationship between two siblings, which affects the reliability index of this factor. Follow-up research can focus on the relationship between two siblings, a stressor that is unique to two-child mothers and set increasingly responsive questions for in-depth investigation.

In summary, the newly compiled questionnaire about the parenting stress experienced by two-child mothers has good reliability and validity and can be used as a tool to measure the parenting stress of two-child mothers. Compared with the existing parenting stress questionnaires, this questionnaire is more suitable for the special group of two-child mothers. The follow-up use of the questionnaire can provide data support in related studies, such as relieving the stress of parenting for two-child mothers and encouraging three-child families.

## Data Availability Statement

The raw data supporting the conclusions of this article will be made available by the authors, without undue reservation.

## Ethics Statement

The studies involving human participants were reviewed and approved by Scientific Research Ethics Committee of Institute of Applied Psychology, Zhejiang University of Technology, China. The patients/participants provided their written informed consent to participate in this study.

## Author Contributions

ZZ and YT designed the study and wrote the manuscript. ZZ, XC, XL, and JT collected the data. ZZ, YT, XC, and XL analyzed the data. All authors contributed to the article and approved the submitted version.

## Conflict of Interest

The authors declare that the research was conducted in the absence of any commercial or financial relationships that could be construed as a potential conflict of interest.

## Publisher’s Note

All claims expressed in this article are solely those of the authors and do not necessarily represent those of their affiliated organizations, or those of the publisher, the editors and the reviewers. Any product that may be evaluated in this article, or claim that may be made by its manufacturer, is not guaranteed or endorsed by the publisher.

## Footnotes

1
https://www.wenjuan.com
