# Supplementary material for: Development and Reliability and Validity Test to the Parenting Stress Questionnaire for Two-Child Mothers
Source: Front Psychol. 2022 May 4;13:850479. doi: 10.3389/fpsyg.2022.850479 (PMC9116054; doi:10.3389/fpsyg.2022.850479)
Supplement: Supplementary file 1 [file Data_Sheet_1.DOCX]

**Table 1 |** The covariance matrix of the item level in subsample 1 (*n* = 279).

|  | **CM 1** | **CM 2** | **CM 3** | **CM 4** | **CM 5** | **CM 6** | **CM 7** | **CM 8** | **CM 9** | **CM 10** | **RS 1** | **RS 2** | **RS 3** | **CC 1** | **CC 2** | **CC 3** | **CC 4** | **EF**  **1** | **EF 2** | **EF 3** | **EF 4** | **EF 5** | **EF 6** | **EF 7** | **EF 8** | **EF 9** |
| --- | --- | --- | --- | --- | --- | --- | --- | --- | --- | --- | --- | --- | --- | --- | --- | --- | --- | --- | --- | --- | --- | --- | --- | --- | --- | --- |
| CM 1 | 1.04 |  |  |  |  |  |  |  |  |  |  |  |  |  |  |  |  |  |  |  |  |  |  |  |  |  |
| CM 2 | 0.59 | 1.34 |  |  |  |  |  |  |  |  |  |  |  |  |  |  |  |  |  |  |  |  |  |  |  |  |
| CM 3 | 0.53 | 0.77 | 1.31 |  |  |  |  |  |  |  |  |  |  |  |  |  |  |  |  |  |  |  |  |  |  |  |
| CM 4 | 0.44 | 0.75 | 0.91 | 1.31 |  |  |  |  |  |  |  |  |  |  |  |  |  |  |  |  |  |  |  |  |  |  |
| CM 5 | 0.47 | 0.70 | 0.63 | 0.58 | 1.28 |  |  |  |  |  |  |  |  |  |  |  |  |  |  |  |  |  |  |  |  |  |
| CM 6 | 0.38 | 0.48 | 0.46 | 0.48 | 0.61 | 1.17 |  |  |  |  |  |  |  |  |  |  |  |  |  |  |  |  |  |  |  |  |
| CM 7 | 0.45 | 0.63 | 0.74 | 0.72 | 0.71 | 0.61 | 1.25 |  |  |  |  |  |  |  |  |  |  |  |  |  |  |  |  |  |  |  |
| CM 8 | 0.49 | 0.61 | 0.73 | 0.80 | 0.70 | 0.62 | 0.97 | 1.47 |  |  |  |  |  |  |  |  |  |  |  |  |  |  |  |  |  |  |
| CM 9 | 0.36 | 0.61 | 0.69 | 0.70 | 0.62 | 0.60 | 0.72 | 0.78 | 1.46 |  |  |  |  |  |  |  |  |  |  |  |  |  |  |  |  |  |
| CM 10 | 0.50 | 0.66 | 0.68 | 0.74 | 0.71 | 0.63 | 0.85 | 0.95 | 0.86 | 1.50 |  |  |  |  |  |  |  |  |  |  |  |  |  |  |  |  |
| RS 1 | 0.16 | 0.26 | 0.27 | 0.25 | 0.31 | 0.24 | 0.32 | 0.26 | 0.17 | 0.27 | 1.01 |  |  |  |  |  |  |  |  |  |  |  |  |  |  |  |
| RS 2 | 0.22 | 0.31 | 0.46 | 0.48 | 0.38 | 0.31 | 0.50 | 0.52 | 0.49 | 0.49 | 0.59 | 1.32 |  |  |  |  |  |  |  |  |  |  |  |  |  |  |
| RS 3 | 0.45 | 0.61 | 0.58 | 0.51 | 0.61 | 0.39 | 0.49 | 0.62 | 0.69 | 0.60 | 0.32 | 0.72 | 1.45 |  |  |  |  |  |  |  |  |  |  |  |  |  |
| CC 1 | 0.36 | 0.35 | 0.50 | 0.48 | 0.41 | 0.32 | 0.49 | 0.57 | 0.58 | 0.49 | 0.17 | 0.47 | 0.69 | 1.45 |  |  |  |  |  |  |  |  |  |  |  |  |
| CC 2 | 0.34 | 0.40 | 0.50 | 0.46 | 0.42 | 0.29 | 0.44 | 0.51 | 0.67 | 0.46 | 0.23 | 0.51 | 0.78 | 0.96 | 1.30 |  |  |  |  |  |  |  |  |  |  |  |
| CC 3 | 0.34 | 0.33 | 0.54 | 0.48 | 0.34 | 0.44 | 0.37 | 0.45 | 0.49 | 0.44 | 0.32 | 0.54 | 0.55 | 0.58 | 0.46 | 1.35 |  |  |  |  |  |  |  |  |  |  |
| CC 4 | 0.37 | 0.43 | 0.50 | 0.48 | 0.39 | 0.30 | 0.47 | 0.54 | 0.48 | 0.35 | 0.15 | 0.38 | 0.65 | 0.62 | 0.68 | 0.55 | 1.39 |  |  |  |  |  |  |  |  |  |
| EF 1 | 0.44 | 0.59 | 0.65 | 0.61 | 0.53 | 0.52 | 0.61 | 0.59 | 0.57 | 0.51 | 0.29 | 0.38 | 0.56 | 0.63 | 0.62 | 0.56 | 0.64 | 1.37 |  |  |  |  |  |  |  |  |
| EF 2 | 0.45 | 0.52 | 0.60 | 0.61 | 0.44 | 0.40 | 0.55 | 0.56 | 0.50 | 0.56 | 0.28 | 0.41 | 0.45 | 0.55 | 0.49 | 0.52 | 0.36 | 0.75 | 1.23 |  |  |  |  |  |  |  |
| EF 3 | 0.41 | 0.48 | 0.49 | 0.54 | 0.49 | 0.43 | 0.49 | 0.51 | 0.51 | 0.51 | 0.22 | 0.36 | 0.36 | 0.55 | 0.46 | 0.51 | 0.42 | 0.90 | 0.72 | 1.23 |  |  |  |  |  |  |
| EF 4 | 0.37 | 0.52 | 0.59 | 0.52 | 0.65 | 0.44 | 0.54 | 0.52 | 0.56 | 0.51 | 0.31 | 0.47 | 0.51 | 0.51 | 0.47 | 0.51 | 0.40 | 0.67 | 0.65 | 0.69 | 1.20 |  |  |  |  |  |
| EF 5 | 0.46 | 0.57 | 0.59 | 0.63 | 0.66 | 0.45 | 0.73 | 0.81 | 0.77 | 0.84 | 0.22 | 0.43 | 0.57 | 0.61 | 0.60 | 0.44 | 0.53 | 0.62 | 0.64 | 0.62 | 0.77 | 1.57 |  |  |  |  |
|  | **CM 1** | **CM 2** | **CM 3** | **CM 4** | **CM 5** | **CM 6** | **CM 7** | **CM 8** | **CM 9** | **CM 10** | **RS 1** | **RS 2** | **RS 3** | **CC 1** | **CC 2** | **CC 3** | **CC 4** | **EF**  **1** | **EF 2** | **EF 3** | **EF 4** | **EF 5** | **EF 6** | **EF 7** | **EF 8** | **EF 9** |
| EF 6 | 0.38 | 0.44 | 0.59 | 0.47 | 0.50 | 0.31 | 0.45 | 0.55 | 0.44 | 0.54 | 0.31 | 0.44 | 0.40 | 0.44 | 0.44 | 0.49 | 0.38 | 0.68 | 0.62 | 0.68 | 0.70 | 0.61 | 1.27 |  |  |  |
| EF 7 | 0.40 | 0.55 | 0.55 | 0.53 | 0.44 | 0.40 | 0.50 | 0.58 | 0.53 | 0.54 | 0.25 | 0.48 | 0.51 | 0.50 | 0.48 | 0.51 | 0.52 | 0.76 | 0.61 | 0.79 | 0.62 | 0.72 | 0.79 | 1.30 |  |  |
| EF 8 | 0.40 | 0.47 | 0.61 | 0.54 | 0.49 | 0.32 | 0.54 | 0.55 | 0.39 | 0.48 | 0.35 | 0.57 | 0.50 | 0.55 | 0.50 | 0.52 | 0.50 | 0.77 | 0.65 | 0.75 | 0.61 | 0.58 | 0.73 | 0.81 | 1.30 |  |
| EF 9 | 0.42 | 0.55 | 0.63 | 0.62 | 0.58 | 0.46 | 0.54 | 0.62 | 0.58 | 0.56 | 0.32 | 0.53 | 0.60 | 0.64 | 0.63 | 0.61 | 0.63 | 0.79 | 0.61 | 0.74 | 0.73 | 0.79 | 0.69 | 0.84 | 0.80 | 1.35 |

*Note*. *CM, Characteristics of Mother; EF, Environmental Factor; CC, Characteristics of Child; RS, Relationship between Two Siblings.*

**The outline of initial interview**

Hello, thank you very much for taking the time to participate in this interview. This is a survey of the parenting pressure of two-child mothers. We want to get real feedback from you. For the need of the study, the entire interview process will be recorded, but please rest assured that all your information will be kept strictly and used only for this study. During the interview process, you can speak freely. Our interview begins right now.

I. Basic personal information

1. First of all, I want to ask for some basic information? What's your date of birth? What are the birth dates of your two children?

II. Interview content

- 1. It is uneasy to raise a second child. After your second child born, do you think your physical and mental state has changed compared with before?
  2. Do you think raising a second child will affect your arrangement for your own development? Like work or private time. What is the specific impact?
  3. Can you talk about what is the division of labor between family members when raising the child? Are you satisfied with this division of labor?
  4. Do you think your husband can share a good job for you in raising a second child? Are you satisfied with his performance?
  5. How about the quality of the relationship between you and other family members in raising your second child?
  6. Does raising a second child affect your interpersonal relationships?
  7. Are there any changes in your family's quality of life before and after raising a second child? Does this confuse you?
  8. Do you feel stressful in terms of children's education? What are the details about it?
  9. Do you have concerns about the future of your two children? What are the specific performance?
  10. Do you think the two children are easy to take care of? What are their own characteristics?
  11. Do you think you feel comfortable dealing with the relationship with your two children?
  12. What is the relationship between the two children? Does the relationship between them often haunt you?
  13. What do you think is the difference between raising a second child and the first child? Especially in terms of the stress that you feel.
  14. In addition to what we just talked about, what else did you feel stressful when you were raising a second child?
  15. Would you like to continue to have a third child? Why ?
  16. Do you have anything else you want to say to us?

Thank you for your participation and cooperation! Have a happy life. Hopefully, you will also support our possible follow-up investigation.

**
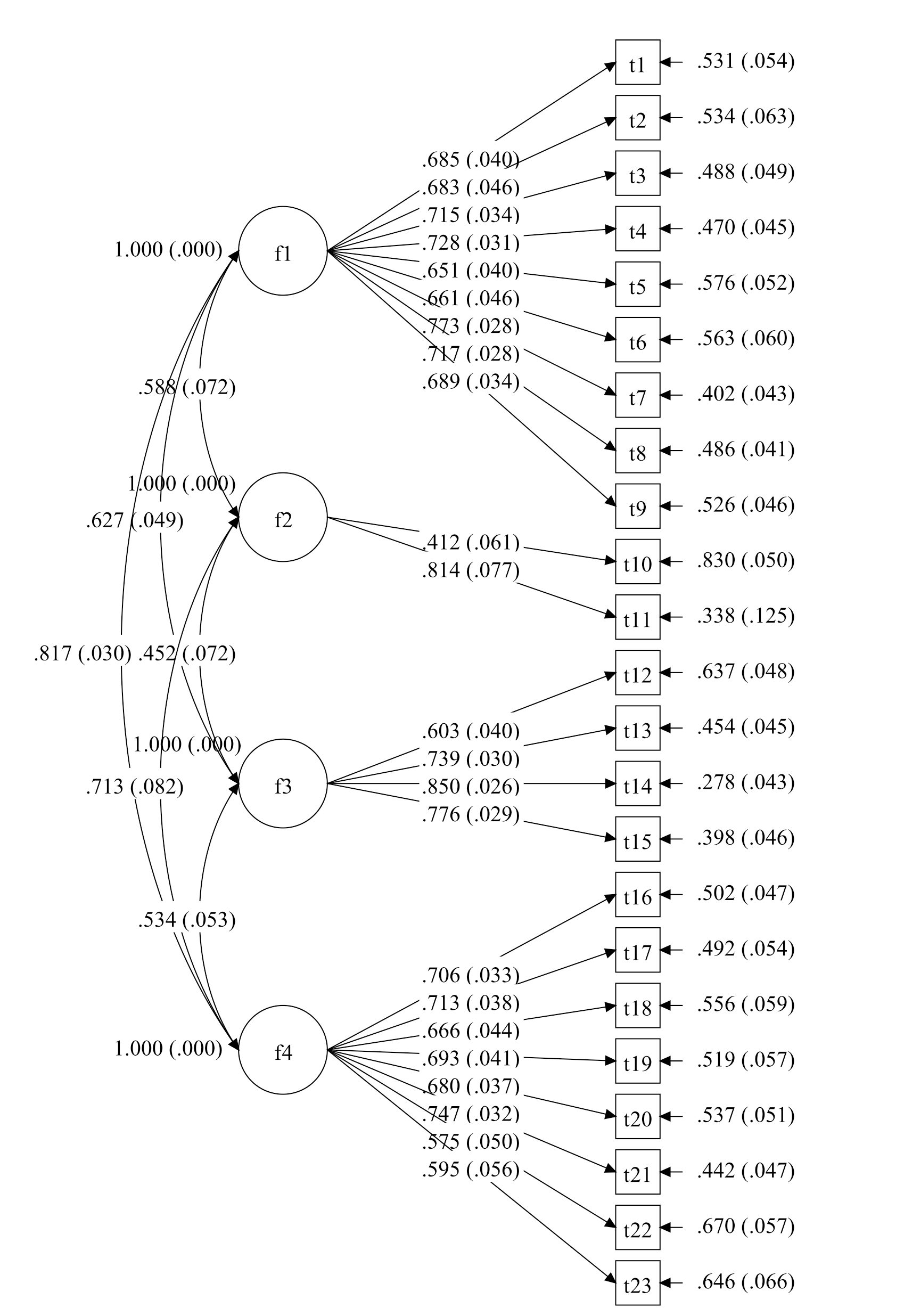
**

**Figure 1 |** Loadings of the four factors in CFA in subsample2 (*n* = 263).

*Note*. *f1, Characteristics of Mother; f2, Relationship between Two Siblings; f3, Characteristics of Child; f4, Environmental Factor.*
